# The chloroplast genome of *Cerasus campanulata* (Maxim.) A.N. Vassiljeva

**DOI:** 10.1080/23802359.2018.1437799

**Published:** 2018-02-12

**Authors:** Tielong Cheng, Yuhao Weng, Liming Yang, Lu Lu, Zhaodong Hao, Jisen Shi, Jinhui Chen

**Affiliations:** a College of Biology and the Environment, Nanjing Forestry University, Nanjing, China;; b Co-Innovation Center for the Sustainable Forestry in Southern China, Nanjing, China;; c Key Laboratory of Forest Genetics and Biotechnology, Ministry of Education, Nanjing Forestry University, Nanjing, China

**Keywords:** *Cerasus campanulata*, chloroplast DNA, genome, phylogeny

## Abstract

The complete chloroplast genome of *Cerasus campanulata* was obtained by using 454 pyrosequencing technology. *Cerasus campanulata* chloroplast genome is 157,906 base pairs containing 115 unique genes, including 79 protein-coding genes, 39 tRNAs and eight rRNAs. Phylogenetic analysis of the protein-coding genes indicates that *C. campanulata* is clearly a member of the Rosaceae order.

*Cerasus campanulata* (Maxim.) A.N. Vassiljeva is a species of cherry that belongs to the Prunoideae focke, a subfamily of the flowering plant Rosaceae order. *C. campanulata* occurs in the Fujian, Guangxi, Guangdong, Hainan and Zhenjiang provinces in China, as well as in Vietnam and Japan (Brach and Song 2006). It usually grows in or on the edge of forests and valleys at 100–1300 m altitude, and flowers in early spring with bright colours and can be cultivated in S, E China. *C. campanulata* has a high ornamental value as the first to flower *Cerasus* species with gaudiness and discernible beauty. *Cerasus campanulata* has high prospects to be further developed for tree cultivation.

Chloroplasts are plant-specific organelles which perform photosynthesis to provide essential energy for plants to synthesize starch, amino acids, pigments and fatty acids (Neuhaus and Emes 2000). Chloroplast genomes supply valuable genetic information for evolutionary and functional studies in plants (Shi et al. 2012). In this study, we conducted the sequencing of *C. campanulata* chloroplast genome, and the phytogentics analysis with other Prunoideae chloroplast genomes.

Our pattern specimens were taken from Yangkou Forest Farm, Fujian, China, located at 117.3O–l18.14E, 26.39–27.12 N. Chloroplast DNA (cpDNA) extraction was conducted using the method reported by Vieira et al. (2014), and the extracted cpDNA was kept in Key Laboratory of Forest Genetics and Biotechnology, Nanjing Forestry University. The complete chloroplast genome of *C. campanulata* was sequenced using 454 pyrosequencing technology. Data analysis found that the chloroplast genome of *C. campanulata* is 157,906 bp in size, with a typical quadripartite structure: a pair of IR regions of 26,432 bp separate the LSC region of 85,929 bp and SSC region of 19,113 bp. The GC content is 36.7%, while the IR region has a GC content of 42.5%, significantly higher than that of the LSC and SSC regions, which are 34.6% and 30.2%, respectively. This is a sequence attribute that is seen more often in published Eurosids chloroplast genomes (Cho et al. 2016; Feng et al. 2017; Gichira et al. 2017). Furthermore, while most repeats in chloroplast genomes are AT rich, a low number of repeats occur within the IR regions.

The complete chloroplast genome of *C. campanulata* contains 115 unique genes, including 79 protein-coding genes. We downloaded the sequences of 62 protein-coding genes from 57 plant species having this same set from the GenBank database, and performed both maximum-likelihood (ML) and maximum parsimony (MP) phylogenetic analyses, which were performed with MEGA7 using a few Prunoideae species. Fifty-seven species which have the same set of 62 protein-codings were used to identify the phylogenetic position of *C. campanulata* by Bayesian inference. Phylogenetic analysis (Figure 1) showed these species were divided into three major clades, comprising the Gymnospermae, Euasterids and Eurosids. *Cerasus campanulata* is a member of subsequently the *Cerasus*, Prunoideae and Rosaceae, within the Eurosids.

**Figure 1.**
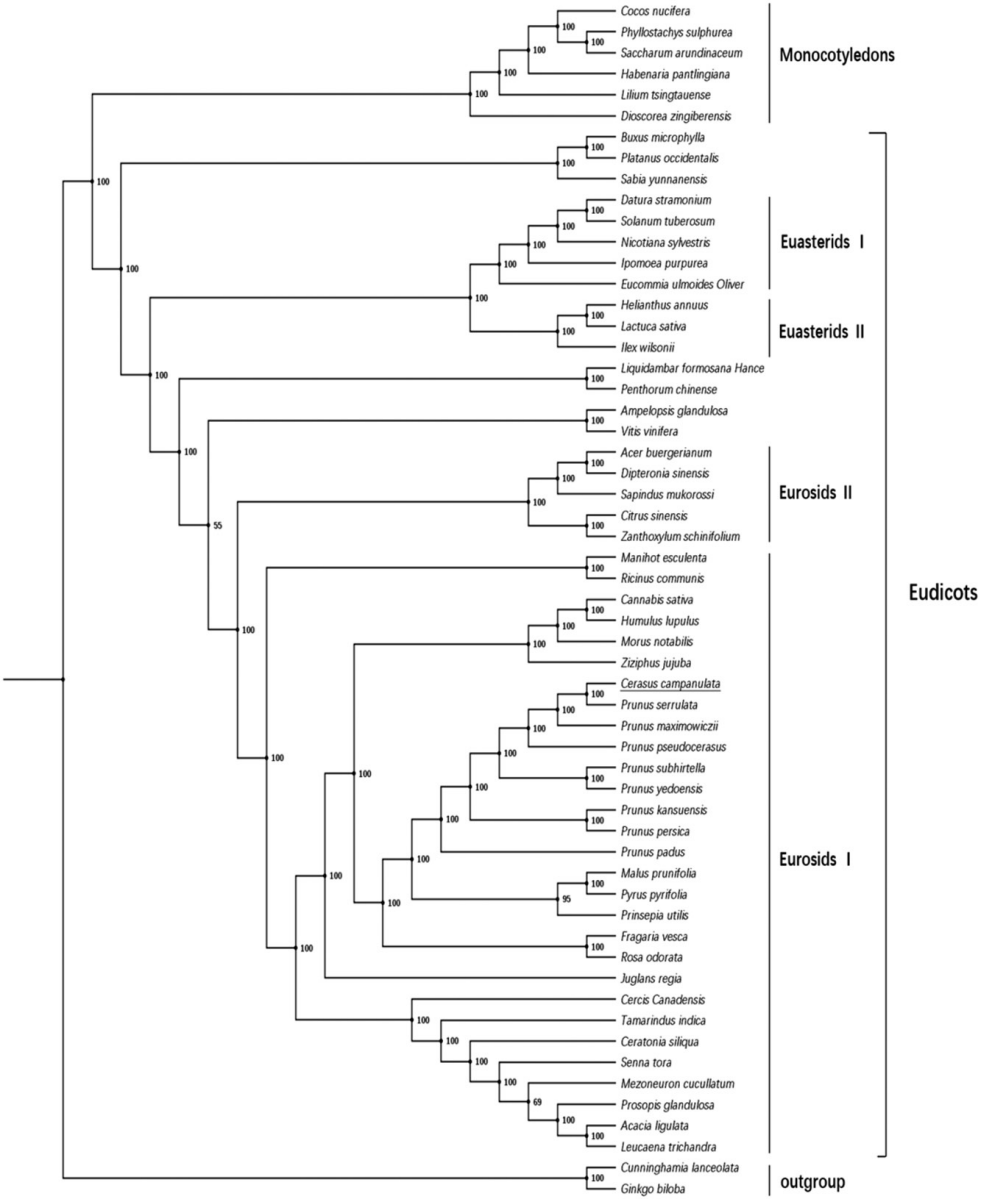
Phylogenetic relationships based on the translated sequences of protein-coding genes. Numbers near the nodes are bootstrap support values. All 57 species’ GenBank accession numbers are listed as below: *Acacia ligulata*, NC_026134.2, *Acer buergerianum*, KF753631.1; *Ampelopsis glandulosa*, KT831767.1; *Buxus microphylla*, EF380351.1; *Cannabis sativa*, NC_026562.1; *Ceratonia siliqua*, KJ468096.1; *Cercis canadensis*, KF856619.1; *Citrus sinensis*, DQ864733.1; *Cocos nucifera*, NC_022417.1; *Cunninghamia lanceolata*, NC_021437.1; *Datura stramonium*, JN654342.1; *Dioscorea zingiberensis*, KP899622.1; *Dipteronia sinensis*, NC_029338.1; *Eucommia ulmoides*, KU204775.1; *Fragaria vesca*, JF345175.1; *Ginkgo biloba*, NC_016986.1; *Habenaria pantlingiana*, NC_026775.1; *Helianthus annuus*, NC_007977.1; *Humulus lupulus*, NC_028032.1; *Ilex wilsonii*, KX426471.1; *Ipomoea purpurea*, EU118126.1; *Juglans regia*, KT870116.1; *Lactuca sativa*, AP007232.1; *Leucaena trichandra*, NC_028733.1; *Lilium tsingtauense*, KU230438.1; *Liquidambar formosana*, NC_023092.1; *Malus prunifolia*, NC_031163.1; *Manihot esculenta*, NC_010433.1; *Mezoneuron cucullatum*, KU569489.1; *Morus notabilis*, NC_027110.1; *Nicotiana sylvestris*, AB237912.1; *Penthorum chinense*, NC_023086.1; *Phyllostachys sulphurea*, NC_024669.1; *Platanus occidentalis*, DQ923116.1; *Prinsepia utilis*, KC571835.1; *Prosopis glandulosa*, KJ468101.1; *Prunus kansuensis*, NC_023956.1; *Prunus maximowiczii*, KP760071.1; *Prunus padus*, KP760072.1; *Prunus persica*, HQ336405.1; *Prunus pseudocerasus*, NC_030599.1; *Prunus serrulata*, KP760073.1; *Prunus subhirtella*, KP760075.1; *Prunus yedoensis*, KP732472.1; *Pyrus pyrifolia*, AP012207.1; *Ricinus communis*, JF937588.1; *Rosa odorata*, KF753637.1; *Sabia yunnanensis*, NC_029431.1; *Saccharum arundinaceum*, NC_030777.1; *Sapindus mukorossi*, NC_025554.1; *Senna tora*, NC_030193.1; *Solanum tuberosum*, NC_008096.2; *Tamarindus indica*, KJ468103.1; *Vaccinium macrocarpon*, JQ248601.1; *Vitis vinifera*, NC_007957.1; *Zanthoxylum schinifolium*, KT321318.1; *Ziziphus jujuba*, NC_030299.1.
